# Supplementary figures and images for: Beyond antibodies: Beta-2 glycoprotein I as the unsung guardian of pregnancy
Source: PLoS One. 2025 Apr 25;20(4):e0321405. doi: 10.1371/journal.pone.0321405 (PMC12027016; doi:10.1371/journal.pone.0321405)

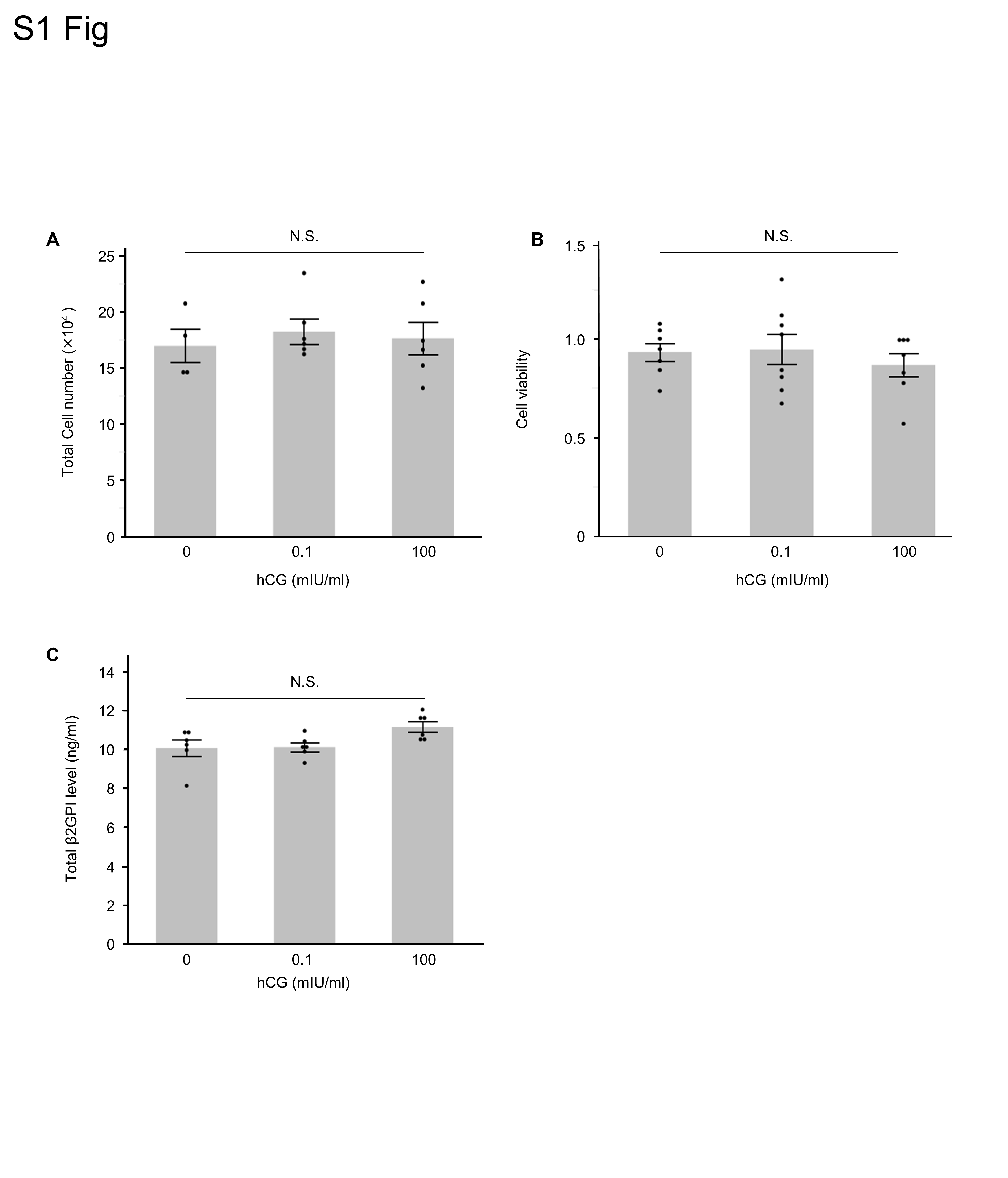

Supplement: S1 Fig — An assessment of (A) the total cell number, (B) CCK-8 assay, and (C) total-β2GPI levels in culture supernatants was performed 24 hours after the addition of hCG (0.1 and 100 mIU/ml). All bars represent the mean ± SEM. Dot plots for each measurement value were superimposed onto the bar graph. Data were evaluated by one-way ANOVA with Shapiro–Wilk normality test and Brown–Forsythe test, followed by Student-Newman-Keuls multiple comparison test. (TIF) [file pone.0321405.s001.TIF]

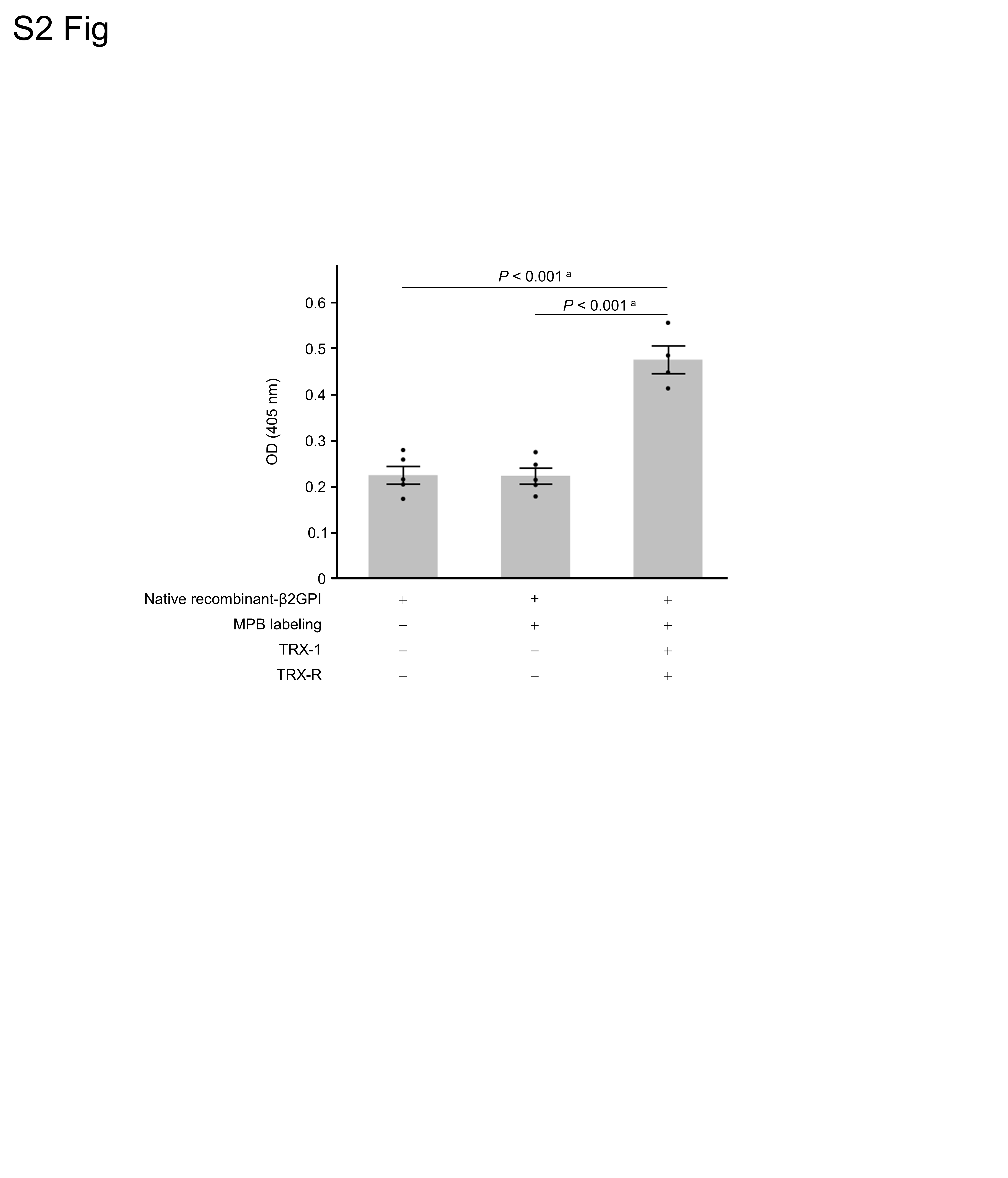

Supplement: S2 Fig — After labeling with the free thiol-specific biotinylated probe, Nα-(3-maleimidylpropionyl) biocytin (MPB), recombinant β2GPI with or without the pretreatment of thioredoxin-1 (TRX-1) and thioredoxin reductase (TRX-R) were incubated on a streptavidin plate and probed with an anti-β2GPI antibody. After washing plates, alkaline phosphatase-conjugated goat anti-rabbit IgG was added, and optical density was read at 405 nm after the addition of a chromogenic substrate. Data were evaluated by one-way ANOVA with Shapiro–Wilk normality test and Brown–Forsythe test, followed by Student-Newman-Keuls multiple comparison test. (TIF) [file pone.0321405.s002.TIF]

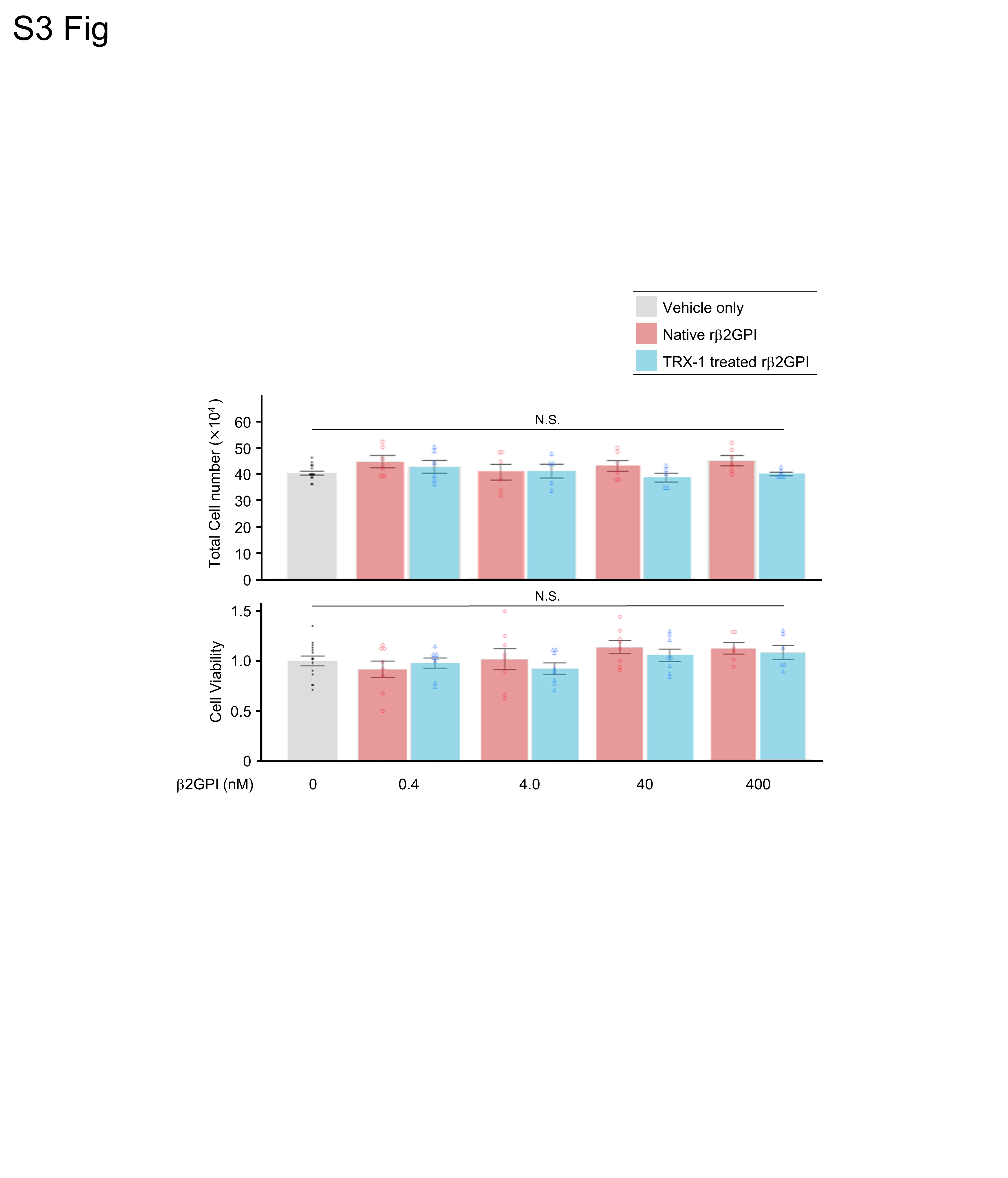

Supplement: S3 Fig — HTR-8/SVneo trophoblast cells were incubated for 24 hours with different concentrations (0, 0.4, 4.0, 40, or 400 nM) of native recombinant β2GPI (rβ2GPI), most of which was in its oxidized form (See S2 Fig), or TRX-1-treated rβ2GPI, which generates free thiols within β2GPI (reduced-β2GPI). (A) Total cell number (n = 6) and (B) cell viability (n = 6) were assessed. All bars represent the mean ± SEM. Dot plots for each measurement value were superimposed onto the bar graph. Data were evaluated by one-way ANOVA with Shapiro–Wilk normality test and Brown–Forsythe test, followed by Student-Newman-Keuls multiple comparison test. (TIF) [file pone.0321405.s003.TIF]
