# Supplementary material for: Beyond antibodies: Beta-2 glycoprotein I as the unsung guardian of pregnancy
Source: PLoS One. 2025 Apr 25;20(4):e0321405. doi: 10.1371/journal.pone.0321405 (PMC12027016; doi:10.1371/journal.pone.0321405)
Supplement: Attachment — Submitted filename: Reviewers comments.docx [file pone.0321405.s005.docx]

**Reviewer’s comments**

A manuscript by Nakamura and co-authors is potentially interesting. It brings a new perspective to the involvement of β2GPI in trophoblast cell function. However, the sudy has some major issues that need to be addressed.

- No details have been given for the Ethical approval for first trimester placental tissue, but only for blood samples. Please give details on who issued the approval, and the approval number for the tissue.
- Materials and Methods section needs more detail on the procedures. For example, labeling of proteins was not described well. Were these proteins from cell culture supernatants? Because the line 153 says “the same pooled serum was used as positive control”. Same to what? It is unclear.
- Only the method for cell viability was given (CCK8), and none for cell number determination, Yet, these data are presented in the Figure 1.
- Another important issue refers to the proliferation of primary trophoblast. In usual cell culture settings, cytotrophoblast does not proliferate, but rather differentiate and fuse in syncitium. Thus, it is unclear how the authors observed the proliferationin primary trophoblast cells.
- Students t-test is not apropriate for multiple compairsons. The results should be analyzed by One-Way ANOVA, followed by appropriate post-hoc test. How were the significances obtained?The authors talk about the dose -dependence (line 263). However, the statistical differences between the treatment groups is not shown, only compared to the control.

Other remarks:

In the Abstract, it is not clearly written what was investigated in the study. A sentence od two should be added to explain what was investigated and why, before the results.

Line 111 – extravillous trophoblast cell line

Line 158 and 159 – give the full names before introducing abbrevations TRX-1 and TRX-R, and briefly explain why they are added.

Line 132 – The title is not correct, no cell proliferation method is described. It is also gramatically not correct.

To what section do lines 389-394 belong? Should they be before Figure 5 lengend? This way this paragraph stands unlinked to other text.
