# Supplementary material for: Beyond antibodies: Beta-2 glycoprotein I as the unsung guardian of pregnancy
Source: PLoS One. 2025 Apr 25;20(4):e0321405. doi: 10.1371/journal.pone.0321405 (PMC12027016; doi:10.1371/journal.pone.0321405)
Supplement: Attachment — Submitted filename: letter for revision_30jan2025.docx [file pone.0321405.s006.docx]

Professor María Teresa Llinás
Academic Editor
PLOS ONE

RE: PONE-D-24-44937

Dear Professor Llinás,

We would like to re-submit our manuscript "**Beyond antibodies: Beta-2 glycoprotein I as the unsung guardian of pregnancy**" for your consideration for publication in *PLOS ONE*.

We appreciate all your comments, which we have read carefully and found very helpful for improving our manuscript. As per your comments, we have revised it for your consideration. The changes are highlighted in yellow colour in the revised manuscript.

We used grants from the Japan Society for the Promotion of Science JSPS for this research project. However, the funders had no role in study design, data collection and analysis, decision to publish, or preparation of the manuscript.

Please find below the list of our alterations and replies to all of your comments.

We believe our manuscript meets the scope and worthiness for publication in *PLOS ONE*. We declare that this manuscript is not submitted elsewhere and we have no conflicts of interest.

We are looking forward to hearing from you soon.

Sincerely yours,

Hitomi Nakamura

Department of Obstetrics and Gynecology,

Osaka University Graduate School of Medicine.

2-2, Yamadaoka, Suita, Osaka 5650871, Japan.

Tel: +81-6-6879-3351

Fax: +81-6-6879-3359

E-mail: hitomi@gyne.med.osaka-u.ac.jp

PONE-D-24-44937

Beyond antibodies: Beta-2 glycoprotein I as the unsung guardian of pregnancy

PLOS ONE

Dear Dr. Nakamura,

Thank you for submitting your manuscript to PLOS ONE. After careful consideration, we feel that it has merit but does not fully meet PLOS ONE’s publication criteria as it currently stands. Therefore, we invite you to submit a revised version of the manuscript that addresses the points raised during the review process.

While the reviewers provided differing perspectives on the extent of the revisions required, we value the constructive feedback from both. We believe that addressing the concerns raised by Reviewer 2, in addition to incorporating Reviewer 1 suggestions, will significantly enhance the manuscript and ensure its suitability for our journal.

Please submit your revised manuscript by Jan 31 2025 11:59PM. If you will need more time than this to complete your revisions, please reply to this message or contact the journal office at [plosone@plos.org](mailto:plosone@plos.org). When you're ready to submit your revision, log on to <https://www.editorialmanager.com/pone/> and select the 'Submissions Needing Revision' folder to locate your manuscript file.

Please include the following items when submitting your revised manuscript:

- A rebuttal letter that responds to each point raised by the academic editor and reviewer(s). You should upload this letter as a separate file labeled 'Response to Reviewers'.
- A marked-up copy of your manuscript that highlights changes made to the original version. You should upload this as a separate file labeled 'Revised Manuscript with Track Changes'.
- An unmarked version of your revised paper without tracked changes. You should upload this as a separate file labeled 'Manuscript'.

If you would like to make changes to your financial disclosure, please include your updated statement in your cover letter. Guidelines for resubmitting your figure files are available below the reviewer comments at the end of this letter.

If applicable, we recommend that you deposit your laboratory protocols in protocols.io to enhance the reproducibility of your results. Protocols.io assigns your protocol its own identifier (DOI) so that it can be cited independently in the future. For instructions see: [https://journals.plos.org/plosone/s/submission-guidelines#loc-laboratory-protocols](https://track.editorialmanager.com/CL0/https:%2F%2Fjournals.plos.org%2Fplosone%2Fs%2Fsubmission-guidelines%23loc-laboratory-protocols/1/010f0193d7375e6c-5241a3c6-7c98-42b8-b199-dec097462b62-000000/mNt__9h4mUbdTkiWEned82X6-SmaS2WrisLcTYU1rII=189). Additionally, PLOS ONE offers an option for publishing peer-reviewed Lab Protocol articles, which describe protocols hosted on protocols.io. Read more information on sharing protocols at [https://plos.org/protocols?utm_medium=editorial-email&utm_source=authorletters&utm_campaign=protocols](https://track.editorialmanager.com/CL0/https:%2F%2Fplos.org%2Fprotocols%3Futm_medium=editorial-email%26utm_source=authorletters%26utm_campaign=protocols/1/010f0193d7375e6c-5241a3c6-7c98-42b8-b199-dec097462b62-000000/dLJBsBH8kYAyqptoFfBCOr8SOSjhamM8K4DvylMf2KE=189).

We look forward to receiving your revised manuscript.

Kind regards,

María Teresa Llinás
Academic Editor
PLOS ONE

Journal requirements:

When submitting your revision, we need you to address these additional requirements.

1. Please ensure that your manuscript meets PLOS ONE's style requirements, including those for file naming. The PLOS ONE style templates can be found at

<https://journals.plos.org/plosone/s/file?id=wjVg/PLOSOne_formatting_sample_main_body.pdf>

and

[https://journals.plos.org/plosone/s/file?id=ba62/PLOSOne_formatting_sample_title_authors_affiliations.pdf](https://track.editorialmanager.com/CL0/https:%2F%2Fjournals.plos.org%2Fplosone%2Fs%2Ffile%3Fid=ba62%2FPLOSOne_formatting_sample_title_authors_affiliations.pdf/1/010f0193d7375e6c-5241a3c6-7c98-42b8-b199-dec097462b62-000000/-gz-eijEyI3LxhbfjkPoMpw_HRAiWe9u-kqCSoZ_7I8=189)

We have read these 2 PLOS ONE’s style templates and have also reviewed the Submission Guidelines at

<https://journals.plos.org/plosone/s/submission-guidelines>.

We reported exact P-values for all values greater than or equal to 0.001. We have checked that the manuscript conforms to PLOS ONE style requirements, including file naming.

2. Thank you for stating the following financial disclosure:

“HN, KM, TM, ME, TK.

The Japan Society for the Promotion of Science JSPS KAKENHI Grant (No. 19K09779, 21K09447, 22K09570) from the Ministry of Education, Science, and Culture of Japan (Tokyo, Japan).”

Please state what role the funders took in the study.  If the funders had no role, please state: "The funders had no role in study design, data collection and analysis, decision to publish, or preparation of the manuscript."

If this statement is not correct you must amend it as needed.

Please include this amended Role of Funder statement in your cover letter; we will change the online submission form on your behalf.

The funders had no role. In the cover letter we state that "The funders had no role in study design, data collection and analysis, decision to publish, or preparation of the manuscript." Could you please change the condition on the web page?

3. Thank you for stating the following in the Acknowledgments Section of your manuscript:

“The authors thank Dr Takeshi Hisamatsu (Hisamatsu Maternity Clinic) for organizing the clinical samples. We extend our gratitude to all patients who consented to provide specimens. This work was supported in part by the Japan Society for the Promotion of Science JSPS KAKENHI Grant (No. 19K09779, 21K09447, 22K09570) from the Ministry of Education, Science, and Culture of Japan (Tokyo, Japan).”

We note that you have provided funding information that is currently declared in your Funding Statement. However, funding information should not appear in the Acknowledgments section or other areas of your manuscript. We will only publish funding information present in the Funding Statement section of the online submission form.

Please remove any funding-related text from the manuscript and let us know how you would like to update your Funding Statement. Currently, your Funding Statement reads as follows:

“HN, KM, TM, ME, TK.

The Japan Society for the Promotion of Science JSPS KAKENHI Grant (No. 19K09779, 21K09447, 22K09570) from the Ministry of Education, Science, and Culture of Japan (Tokyo, Japan).”

Please include your amended statements within your cover letter; we will change the online submission form on your behalf.

(Acknowledgments, line 500-502) We have removed “This work was supported in part by the Japan Society for the Promotion of Science JSPS KAKENHI Grant (No. 19K09779, 21K09447, 22K09570) from the Ministry of Education, Science, and Culture of Japan (Tokyo, Japan).”

4. We note that your Data Availability Statement is currently as follows: [All relevant data are within the manuscript and its Supporting Information files.]

Please confirm at this time whether or not your submission contains all raw data required to replicate the results of your study. Authors must share the “minimal data set” for their submission. PLOS defines the minimal data set to consist of the data required to replicate all study findings reported in the article, as well as related metadata and methods (<https://journals.plos.org/plosone/s/data-availability#loc-minimal-data-set-definition>).

For example, authors should submit the following data:

- The values behind the means, standard deviations and other measures reported;

- The values used to build graphs;

- The points extracted from images for analysis.

Authors do not need to submit their entire data set if only a portion of the data was used in the reported study.

If your submission does not contain these data, please either upload them as Supporting Information files or deposit them to a stable, public repository and provide us with the relevant URLs, DOIs, or accession numbers. For a list of recommended repositories, please see <https://journals.plos.org/plosone/s/recommended-repositories>.

If there are ethical or legal restrictions on sharing a de-identified data set, please explain them in detail (e.g., data contain potentially sensitive information, data are owned by a third-party organization, etc.) and who has imposed them (e.g., an ethics committee). Please also provide contact information for a data access committee, ethics committee, or other institutional body to which data requests may be sent. If data are owned by a third party, please indicate how others may request data access.

We share the “minimal data set” for our submission.

[Note: HTML markup is below. Please do not edit.]

Reviewers' comments:

Reviewer's Responses to Questions

**Comments to the Author**

1. Is the manuscript technically sound, and do the data support the conclusions?

The manuscript must describe a technically sound piece of scientific research with data that supports the conclusions. Experiments must have been conducted rigorously, with appropriate controls, replication, and sample sizes. The conclusions must be drawn appropriately based on the data presented.

Reviewer #1: Partly

Reviewer #2: Yes

2. Has the statistical analysis been performed appropriately and rigorously?

Reviewer #1: No

Reviewer #2: Yes

3. Have the authors made all data underlying the findings in their manuscript fully available?

The [PLOS Data policy](http://track.editorialmanager.com/CL0/http:%2F%2Fwww.plosone.org%2Fstatic%2Fpolicies.action%23sharing/1/010f0193d7375e6c-5241a3c6-7c98-42b8-b199-dec097462b62-000000/peCiFhiHGX_NgF2CtV--6QxFlVQLlPVhcKMKwO9fQQY=189) requires authors to make all data underlying the findings described in their manuscript fully available without restriction, with rare exception (please refer to the Data Availability Statement in the manuscript PDF file). The data should be provided as part of the manuscript or its supporting information, or deposited to a public repository. For example, in addition to summary statistics, the data points behind means, medians and variance measures should be available. If there are restrictions on publicly sharing data—e.g. participant privacy or use of data from a third party—those must be specified.

Reviewer #1: Yes

Reviewer #2: Yes

4. Is the manuscript presented in an intelligible fashion and written in standard English?

PLOS ONE does not copyedit accepted manuscripts, so the language in submitted articles must be clear, correct, and unambiguous. Any typographical or grammatical errors should be corrected at revision, so please note any specific errors here.

Reviewer #1: Yes

Reviewer #2: Yes

5. Review Comments to the Author

Please use the space provided to explain your answers to the questions above. You may also include additional comments for the author, including concerns about dual publication, research ethics, or publication ethics. (Please upload your review as an attachment if it exceeds 20,000 characters)

Reviewer #1:

A manuscript by Nakamura and co-authors is potentially interesting. It brings a new perspective to the involvement of β2GPI in trophoblast cell function. However, the sudy has some major issues that need to be addressed.
- No details have been given for the Ethical approval for first trimester placental tissue, but only for blood samples. Please give details on who issued the approval, and the approval number for the tissue.

We added details for first trimester placenta tissues as below (the changes are highlighted in yellow colour);

(line 185, Study approval) This study was approved by the Institutional Review Board (IRB) of Osaka University Medical School Hospital. Written informed consent was obtained from all patients for providing blood samples using a comprehensive consent form (#11111-4) and for providing first trimester placenta tissues using specific consent form (#20283(T2)). The retrospective study used blood samples and patient information collected and stored after consent was obtained using a comprehensive consent form under IRB approval (#20283(T2)). For the blood samples in this study instead of obtaining informed consent from each patient in accordance with the IRB, opt-out was done over the web.

- Materials and Methods section needs more detail on the procedures. For example, labeling of proteins was not described well. Were these proteins from cell culture supernatants? Because the line 153 says “the same pooled serum was used as positive control”. Same to what? It is unclear.

We have changed as below;

(Line 150 in the revised manuscript with changes) The percentage of reduced (free thiol) β2GPI within the cell culture supernatant and patient samples were ~~was~~ measured as previously described…

(Line 161 in the revised manuscript with changes) As a positive control, we used the same pooled serum from 10 healthy controls for all of the study.

- Only the method for cell viability was given (CCK8), and none for cell number determination, Yet, these data are presented in the Figure 1.

We have added the section “cell counting” as below;

(Line 135 in the revised manuscript with changes)

Cell counting

Ten microliters of cell suspension were analysed using a haemocytometer.

- Another important issue refers to the proliferation of primary trophoblast. In usual cell culture settings, cytotrophoblast does not proliferate, but rather differentiate and fuse in syncitium. Thus, it is unclear how the authors observed the proliferationin primary trophoblast cells.

We have changed our manuscript to mention cell number and CCK-8 assay data instead of using the term "proliferation".

- Students t-test is not apropriate for multiple compairsons. The results should be analyzed by One-Way ANOVA, followed by appropriate post-hoc test. How were the significances obtained?The authors talk about the dose -dependence (line 263). However, the statistical differences between the treatment groups is not shown, only compared to the control.

We have re-analysed data and made some changes in our manuscript as below;

(line 226 in Statistical analysis in Materials and Methods)

“Comparisons among groups were conducted using a one-way ANOVA or Kruskal-Wallis ANOVA on Ranks with Shapiro–Wilk normality test and Brown–Forsythe test, followed by Student-Newman-Keuls multiple comparison test. To assess the difference between the two groups, data were analyzed using the Student’s t-test ~~or Wilcoxon’s rank-sum test~~ with the Shapiro–Wilk normality test.”

Other remarks:
In the Abstract, it is not clearly written what was investigated in the study. A sentence od two should be added to explain what was investigated and why, before the results.

We have added one sentence before explaining our results as below;

(Abstract) “The physiological function of beta 2 glycoprotein I (β2GPI) itself is not well understood, other than that it is a primary antigen to anti-phospholipid antibodies in the autoimmune disease antiphospholipid syndrome. β2GPI is a soluble blood protein that is predominantly synthesized in hepatocytes. Why is the expression of β2GPI observed in the placenta despite its abundance in the circulating blood of healthy individuals? Does the placenta produce a specific-acting β2GPI?

β2GPI was recently shown to adopt two interconvertible biochemical confirmations based on the integrity of disulfide bonds: oxidized and reduced. The present study investigates the physiological function of β2GPI in trophoblast cells, with a focus on the reduced and oxidized forms of β2GPI under the hypothesis that placental β2GPI has a different activity from circulating β2GPI. Endogenous β2GPI secretion in trophoblast cells were predominantly in the reduced form, while those in HepG2 liver cells were mainly in the oxidized form. Progesterone increased reduced-β2GPI in both the trophoblast and liver cells. Oxidized-β2GPI significantly inhibited trophoblast cell migration and increased placental soluble fms-like tyrosine kinase-1 (sFlt-1). Furthermore, excess sFlt-1 significantly increased oxidized-β2GPI secretion in HepG2 cells. Circulating oxidized-β2GPI levels were significantly higher in women with pre-eclampsia than in those without pre-eclampsia. Therefore, oxidized-β2GPI may contribute to the pathogenesis of pre-eclampsia. Under oxidative stress, the excessive oxidation of β2GPI and/or excessive placental sFlt-1 may trigger a negative spiral between trophoblast and liver cells.”

Line 111 – extravillous trophoblast cell line

We have added “extravillous” as below;

(Line 114 in the revised manuscript with changes)

“Human first trimester extravillous trophoblast, HTR-8/SVneo,…”

Line 158 and 159 – give the full names before introducing abbrevations TRX-1 and TRX-R, and briefly explain why they are added.

We have changed as below;

(Line 167 in the revised manuscript with changes)

…was preincubated with thioredoxin-1 (TRX-1) (3.5 μM) activated with thioredoxin reductase (TRX-R) (10 nM) plus nicotinamide adenine dinucleotide phosphate (NADPH; 200 µM) to generate free thiols within β2GPI.

Line 132 – The title is not correct, no cell proliferation method is described. It is also gramatically not correct.
We have deleted “in cell proliferation assays” and added the section “cell counting” as below;

(Line 135 in the revised manuscript with changes)

**Cell counting**

Ten microliters of cell suspension were analysed using a haemocytometer.

To what section do lines 389-394 belong? Should they be before Figure 5 lengend? This way this paragraph stands unlinked to other text.

(Line 407 in the revised manuscript with changes)

We have moved this part, it is now just before the figure legend for Fig 5.

Reviewer #2:

This article in the abstract and introduction sets the scene why this research was undertaken which is easy to follow. The researchers may also wish to briefly include a few lines either in their discussion or introduction the recent online publication in Nature 11 December 2024 by Kelsey L Swingle et al Placenta-tropic VEGF mRNA lipid nanoparticles ameliorate murine pre-eclampsia, which found that "an endogenous targeting mechanism based on β2-glycoprotein I adsorption that enables LNP delivery to the placenta." This seems to further support the hypothesis of this paper that beta 2 glycoprotein I plays an important biological role in placenta biology.
The methods and ethics and statistical methodology are sound
Results figures and tables are easy to follow and have a logical sequence
My only concern is with line 281-282 (an approximately 5 fold increase.. Fig 3A). When I look at Figure 3A the increase is much less than 5 fold, going from approximately 12.5 ng/ml of total B2GPI in control to 20ng/ml of the TRXI-TRXR treated and to 16ng/ml total B2GPI for the pCMV3-APOH without TRX1-TRXR treatment.
The discussion is easy to follow and ties the results together nicely.

It was an honour for us to have our manuscript reviewed by you, as we are admirers of your β2GPI work.

(Line 281-282 in original, Line 304 in the Revised Manuscript with Track Changes)

The error has been corrected as follows.

“The secretion of total-β2GPI significantly increased in the *APOH*-transferred groups (~~an approximately 5-fold increase,~~ *P* = 0.006, *P* = 0.002, without or with TRX-1 treatment group, respectively. vs. control plasmid DNA-transferred group with no TRX treatment, *P* < 0.001, vs. control plasmid DNA-transferred group with TRX-1 treatment group; *~~P~~* ~~< 0.001, Wilcoxon rank-sum test,~~ Fig 3A).”

We mentioned the recent manuscript (Swingle KL et al., Nature 2025 Jan;637:412-421.), which you referred to, in the discussion part as follows;

(line 468 in the Revised Manuscript with Track Changes)

“Very recently, placenta-tropic lipid nanoparticles (LNPs) using a mechanism based on absorption with endogenous β2GPI have been reported [59]. The study demonstrated that the uptake of LNPs in the liver was significantly increased in β2GPI knockdown mice following intravenous siRNA administration, while it was reduced in the placenta [59]. Furthermore, a significant increase in blood β2GPI levels was observed in inflammation-induced PE model mice, and these mice showed a higher uptake of LNPs in the liver but not in the placenta. These results suggest the possibility of differential actions of β2GPI in the placenta and in the liver.”

6. PLOS authors have the option to publish the peer review history of their article ([what does this mean?](https://track.editorialmanager.com/CL0/https:%2F%2Fjournals.plos.org%2Fplosone%2Fs%2Feditorial-and-peer-review-process%23loc-peer-review-history/1/010f0193d7375e6c-5241a3c6-7c98-42b8-b199-dec097462b62-000000/YPdY_-kw7hJ9LvP9l15HLLyaiCaSjy_pxM3MaXnkQgg=189)). If published, this will include your full peer review and any attached files.


If you choose “no”, your identity will remain anonymous but your review may still be made public.


**Do you want your identity to be public for this peer review?** For information about this choice, including consent withdrawal, please see our [Privacy Policy](https://track.editorialmanager.com/CL0/https:%2F%2Fwww.plos.org%2Fprivacy-policy/1/010f0193d7375e6c-5241a3c6-7c98-42b8-b199-dec097462b62-000000/YxHLHovcyNtHHkGcdcsVhc86o_hiOoUvpYaHceL18Xk=189).

Reviewer #1: No

Reviewer #2: **Yes:** Bill Giannakopoulos
